# Structure of the Mg-Chelatase Cofactor GUN4 Reveals a Novel Hand-Shaped Fold for Porphyrin Binding

**DOI:** 10.1371/journal.pbio.0030151

**Published:** 2005-04-26

**Authors:** Mark A Verdecia, Robert M Larkin, Jean-Luc Ferrer, Roland Riek, Joanne Chory, Joseph P Noel

**Affiliations:** **1**Chemical Biology and Proteomics Laboratory, Salk Institute for Biological StudiesLa Jolla, CaliforniaUnited States of America; **2**Howard Hughes Medical Institute, Plant Molecular and Cellular Biology LaboratorySalk Institute for Biological Studies, La Jolla, CaliforniaUnited States of America; **3**The Institut de Biologie StructuraleGrenobleFrance; University of North CarolinaUnited States of America

## Abstract

In plants, the accumulation of the chlorophyll precursor Mg-protoporphyrin IX (Mg-Proto) in the plastid regulates the expression of a number of nuclear genes with functions related to photosynthesis. Analysis of the plastid-to-nucleus signaling activity of Mg-Proto in Arabidopsis thaliana led to the discovery of GUN4, a novel porphyrin-binding protein that also dramatically enhances the activity of Mg-chelatase, the enzyme that synthesizes Mg-Proto. GUN4 may also play a role in both photoprotection and the cellular shuttling of tetrapyrroles. Here we report a 1.78-Å resolution crystal structure of *Synechocystis* GUN4, in which the porphyrin-binding domain adopts a unique three dimensional fold with a “cupped hand” shape. Biophysical and biochemical analyses revealed the specific site of interaction between GUN4 and Mg-Proto and the energetic determinants for the GUN4 • Mg-Proto interaction. Our data support a novel protective function for GUN4 in tetrapyrrole trafficking. The combined structural and energetic analyses presented herein form the physical-chemical basis for understanding GUN4 biological activity, including its role in the stimulation of Mg-chelatase activity, as well as in Mg-Proto retrograde signaling.

## Abstract 2

The structure of GUN4 offers hints for understanding its role in regulating the biosynthesis of chlorophyll

## Introduction

Organelle function is controlled primarily by the regulation of nuclear gene expression in response to developmental and environmental cues. In turn, organelles signal to the nucleus, in a process termed retrograde signaling, to coordinate the biological activities of the two subcellular compartments. For example, in animals and yeast, mitochondria-to-nucleus and ER-to-nucleus signaling have a dramatic impact on cellular activities under a variety of conditions [1,2]. In plants, plastid-to-nucleus signaling significantly alters the expression of nuclear genes that encode chloroplast-localized proteins involved in photosynthesis and leaf morphogenesis [3,4,5,6]. Therefore, signals originating from plastids play major roles in photoautotrophic growth.

Genetic and physiological studies indicate that the accumulation of the chlorophyll precursors Mg-protoporphyrin IX (Mg-Proto) and Mg-protoporphyrin IX monomethyl ester (Mg-ProtoMe) act as a plastid signal that regulates nuclear gene expression in plants and algae [6,7,8,9,10,11]. The current hypothesis proposes that the plastid exports Mg-Proto and/or Mg-ProtoMe, which then interact with a cytoplasmic signaling pathway that ultimately regulates nuclear gene expression [6,9,11]. This proposed model is not without precedent; heme, a tetrapyrrole that bears a striking resemblance to Mg-Proto, regulates gene expression in animal, yeast, and bacterial cells by binding to transcription factors or to kinases that regulate translation [12,13,14,15,16]. Moreover, the bulk of cellular heme is produced in chloroplasts, which is then transported to other cellular compartments [16,17]. Because of the molecular similarity between heme and Mg-Proto, it is reasonable to assume that the cellular machinery used to export heme from the chloroplast may be similar to the machinery used for Mg-Proto export.

In a search for mutants that affect communication between chloroplasts and the nucleus, a number of mutants, called *gun* mutants, were identified that have defects in plastid-to-nucleus signaling pathways. These plastid-to-nucleus signaling pathways repress the transcription of nuclear genes that encode proteins active in photosynthesis when chloroplast development is blocked [18,19]. A number of *GUN* genes were found to encode factors that participate in Mg-Proto metabolism. Among these were subunits of Mg-chelatase, the enzyme that synthesizes Mg-Proto from protoporphyrin IX (Proto), and indeed, we have shown that buildup of Mg-Proto is a signal that regulates nuclear gene expression [6,18]. GUN4 participates in the same Mg-Proto signaling pathway that Mg-chelatase does, but GUN4 is not related to any previously described Mg-chelatase subunit or any gene with a known function [20]. Purification of a GUN4 complex from *Arabidopsis* thylakoids revealed that a fraction of GUN4 is tightly associated with GUN5 [20], also called ChlH, which is the 140-kDa subunit of Mg-chelatase [18,21]. Although GUN4 is not essential for Mg-Proto synthesis in vitro or in *Arabidopsis,* GUN4 is required for chlorophyll accumulation in *Arabidopsis* under normal growth conditions, GUN4 binds porphyrins in vitro, and GUN4 stimulates Mg-chelatase in vitro [20].

Thus, GUN4, like GUN5, is a key participant in the generation of a plastid signal. Also, because GUN4 appears to be monomeric or associated with heterogeneous complexes in fractionated chloroplasts, and because GUN4 binds to Mg-Proto more tightly than GUN5 in *Synechocystis,* it is reasonable to expect that GUN4 might perform additional functions in porphyrin metabolism [20]. For instance, GUN4 might participate in Mg-Proto trafficking or shield Mg-Proto from collisions with molecular oxygen and light, collisions that could result in the production of reactive oxygen species. Alternatively, GUN4 might protect Mg-Proto from catabolic enzymes found in the plastid [22], or it might participate in other tetrapyrrole biosynthetic reactions localized to plastids [23].

To better understand the porphyrin-binding mechanism and Mg-chelatase stimulatory activity of GUN4, we determined the crystal structure of a GUN4 homolog from the model cyanobacterium *Synechocystis* sp. PCC 6803 (hereafter referred to as *Synechocystis* 6803) and refined the three-dimensional model to 1.78 Å resolution. The crystal structure reveals a novel fold that bears no resemblance to known porphyrin-binding proteins. We subsequently used nuclear magnetic resonance (NMR) to map the porphyrin-binding site through analysis of chemical shift data. Quantitative analysis of the putative porphyrin binding site on GUN4 using fluorescence quenching and enzymatic assays has allowed us to determine the energetic contribution of key residues for porphyrin binding, as well as for the enhancement of Mg^2+^ incorporation into metal-free porphyrins. Taken together, these data paint a picture of a novel enzymatic cofactor that enhances Mg-Proto biosynthesis and additionally, may play a role in Mg-Proto shuttling and chemical protection within the cell.

## Results

### Crystallization, Structure Determination, and Refinement

The crystallized protein (residues 1–233) comprises the entire *Synechocystis* GUN4 (SynGUN4) protein. Full-length protein was shown to be competent for binding to both deuteroporphyrin IX (Deutero) and Mg-deuteroporphyrin IX (Mg-Deutero). It was also shown to significantly enhance Mg^2+^ incorporation into Deutero in the presence of the *Synechocystis* Mg-chelatase, which contains the ChlD, ChlH, and ChlI subunits [21]. The SynGUN4 structure was solved by multiple isomorphous replacement using crystals soaked with methyl mercury (II) chloride (Hg) and potassium tetrachloroplatinate II-containing compounds. The resultant model was built and refined to a crystallographic *R*_cryst_ and *R*_free_ of 22.1% and 25.9%, respectively, using all Hg data extending to 1.78 Å resolution (Table 1).

**Table 1:**
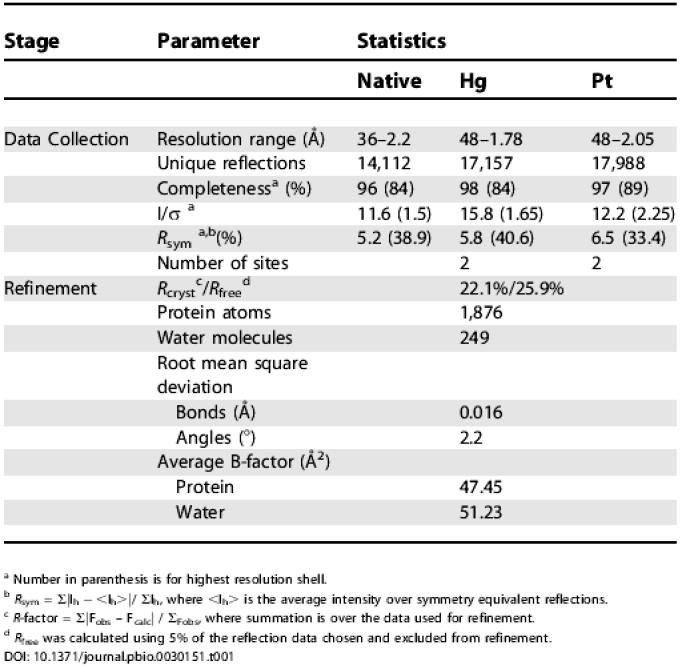
Statistics from the Crystallographic Analysis

^a^ Number in parenthesis is for highest resolution shell

^b^ *R*_sym_ = Σ|I_h_ − <I_h_>|/ ΣI_h_, where <I_h_> is the average intensity over symmetry equivalent reflections

^c^ *R*-factor = Σ|F_obs_ – F_calc_| / Σ_Fobs_, where summation is over the data used for refinement

^d^ *R*_free_ was calculated using 5% of the reflection data chosen and excluded from refinement

### Structure of GUN4

The crystal structure of SynGUN4 reveals a two-domain protein linked by a 12-residue loop (Figure 1A). The C-terminal domain, which we refer to herein as the GUN4 core domain, is the conserved domain among all GUN4 family members. The N-terminal domain of SynGUN4 is composed of the first five helices of the full-length protein. The α1′ to α4′ helices fold into a right-handed, up-and-down helix bundle, and the addition of the α5′ helix gives the domain an elongated cross-section. Overall, the N-terminal helical bundle is reminiscent in appearance to other entirely helical domains such as the TPR domain [24] or the 14–3-3 domain [25]. The entire helical bundle is held together through a hydrophobic core consisting entirely of centrally located and interdigitated leucines, isoleucines, and valines provided by each helix. In contrast to the N-terminal domain's core, the surface is highly charged (Figure 1B). Structurally, the α2′ and α3′ helices are the linchpins of this tertiary architecture as they bridge one end of the bundle to the other.

**Figure 1:**
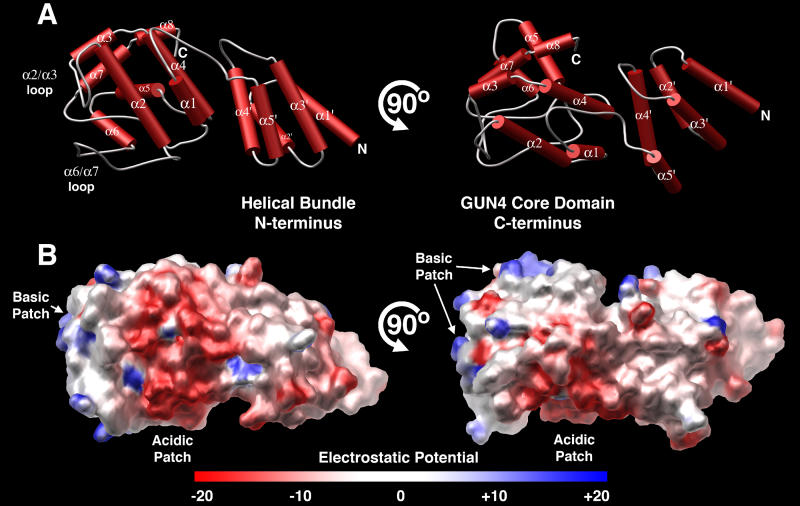
Overall Structure of *Synechocystis* GUN4 (A) Orthogonal views of the crystal structure of the full length (residues 1–233) *Synechocystis* GUN4 protein (SynGUN4). Helices are shown as red cylinders and loop regions are displayed as gray loops. SynGUN4 contains two distinct domains linked by a flexible loop. The helices of the N-terminal domain are labeled with apostrophes to distinguish them from the helices making up the C-terminal domain. All structure figures were made with MOLSCRIPT [57] and POV-Ray (http://www.povray.org). (B) Orthogonal views of the GRASP [58] representation of the SynGUN4 solvent-accessible surface colored to approximately reflect the underlying electrostatic potential, where blue is positive, red is negative, and white is neutral.

Interestingly, analysis of the known GUN4 sequences from other organisms shows that the N-terminal region is the most highly variable sequence and is unique to only some of the prokaryotic family members. SynGUN4's N-terminal region shares homology with GUN4 family members from Gracilaria tenuistipitata var. *liui, Porphyra purpurea, Nostoc* sp. 7120, *Trichodesmium erythraeum, Anabaena variabilis, Thermosynechococcus elongatus,* and Cyanidium caldarium (Figure 2A). The Cyanidioschyzon merolae sequence lacks the N-terminal extension altogether. Additionally, within the *Arabidopsis* and rice GUN4 sequences, the N-terminal domain is replaced with a chloroplast transit peptide, which is removed upon import into the chloroplast [26]. What role this N-terminal helical bundle plays within prokaryotes is unknown, but most likely it does not participate in GUN4 functions conserved between prokaryotic and eukaryotic organisms.

**Figure 2:**
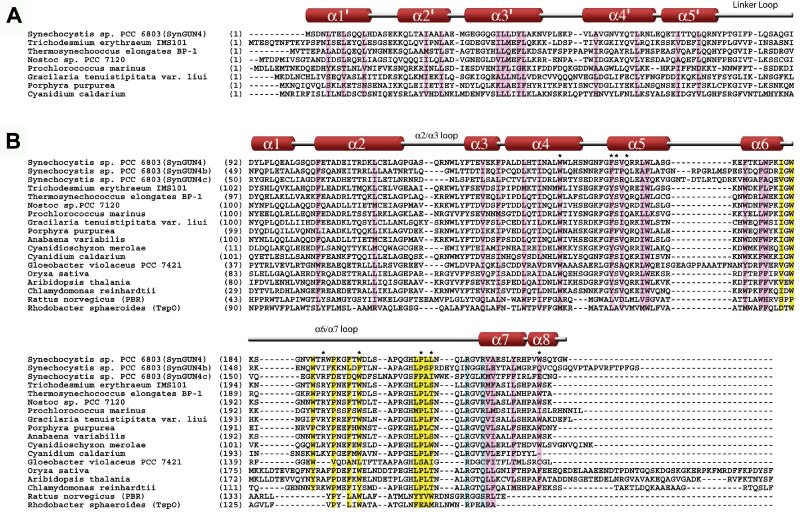
Sequence Alignment of GUN4 and GUN4-like Proteins (A) Alignment of the N-terminal portions of GUN4 family members whose N-terminal domains show sequence homology to SynGUN4. Residues contributing to the hydrophobic core of the five-helix bundle are highlighted (pink). GUN4 sequences isolated from plants thus far all have a plastid transit peptide in place of the N-terminal domain found in SynGUN4. The Chlamydomonas reinhardtii sequence was derived from sequence data produced by the United States Department of Energy Joint Genome Institute (http://www.jgi.doe.gov/). The N-terminal sequence of C. reinhardtii is not yet known but it most likely contains a chloroplast transit peptide. (B) Sequence alignment of possible GUN4 core domains. Residues that form the “palm” of the “cupped hand” are highlighted in pink. Residues from the α6/α7 loop that structure the loop and protrude into the core are highlighted in yellow. Arg214 and Arg217, predicted to be important for binding to porphyrins, are highlighted in blue. Residues that disrupt proper folding when mutated and expressed in E. coli are denoted by an asterisk (*).

The N-terminal domain links to the C-terminal domain through a long loop (residues 81–93) that connects the α5′ helix of the N-terminal helical bundle to the α1 helix within the GUN4 core domain (Figure 2B). The interaction between the two domains is not extensive and is mediated by van der Waal's interactions between Pro51 and Leu53 from the α4′ helix and Trp151 and Leu152 from the α4 helix within the C-terminal domain. Additionally, the carbonyl oxygen of Gly101 on the α1 helix is within hydrogen bonding distance of Asn60 and Arg63 located on the α4′ helix. In all, a total of 1,183 Å^2^ of surface area is buried between the two domains, which suggests that the particular arrangement displayed in the crystal packing may be one of several possible orientations juxtaposing the two domains in solution.

The C-terminal domain of SynGUN4, the GUN4 core domain, appears to have no currently identified structural homologs, as indicated by a lack of any structural matches from a search of the DALI server [27]. The GUN4 core domain maintains two distinct architectural regions: a highly structured helical section that forms the majority of the domain, and two loops (α2/α3 loop and α6/α7 loop), which constitute one side of the domain (Figure 3A). The helices adopt a concave shell shape resembling a “cupped hand” on one side of the domain, with the back of the “hand” facing toward the N-terminal helical bundle. Helices α1 and α2 extend like a thumb and index finger to form one side of the “cupped hand.” The α3 and α4 helices compose the middle finger of the hand, with the α3/α4 loop forming a knuckle. The α5 and α6 and the α7 and α8 helices form the remaining fingers, respectively, on the opposite side of the “cupped hand” arrangement.

**Figure 3:**
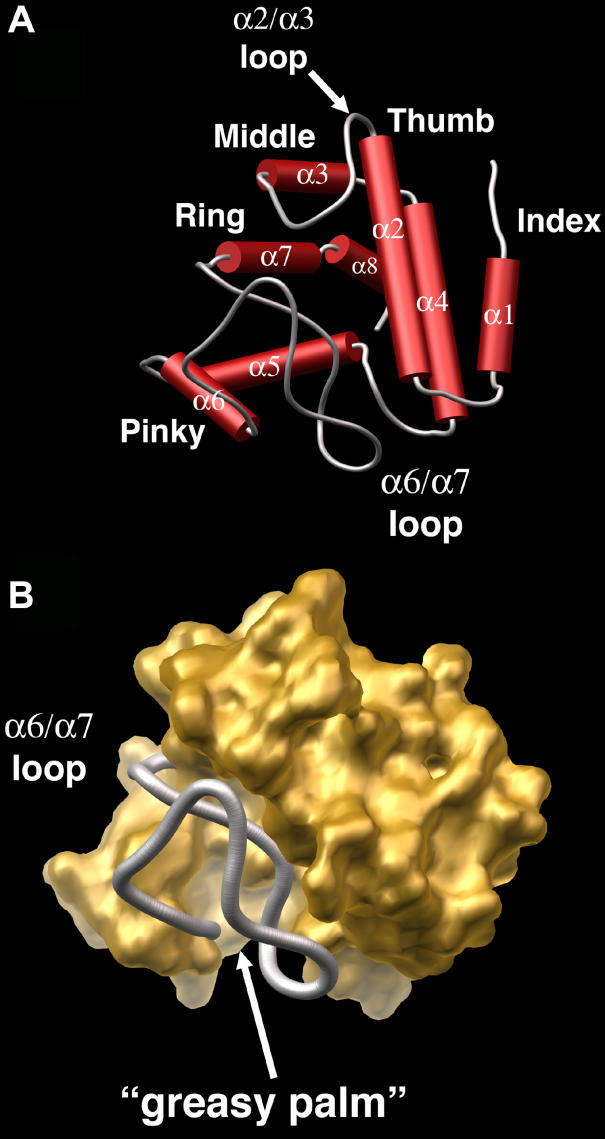
Close-Up View of the GUN4 Core Domain's “Cupped Hand” Architecture (A) Rendered skeletal view of the GUN4 core domain. Helices are shown as red cylinders, and coiled regions are depicted as gray loops. The overall shape resembles that of a “cupped hand.” (B) Rendered view of the solvent-accessible surface of the GUN4 core domain, colored gold. The α6/α7 loop is colored gray and is bound by the remainder of the domain. The “cupped hand” grips this loop.

In total, the helical section constitutes 70% of the GUN4 core domain. The buried surface within the concave section of the “cupped hand” is highly hydrophobic. Phe105 and Leu116 on the α2 helix; Val135 and Phe138 from the α3 helix; Leu143, Ile146, and Trp150 from the α4 helix; Phe160, Val162, Val166, and Trp167 from the α5 helix; Phe174, Leu177, and Trp178 from the α6 helix; Val218, Ala219, and Tyr223 from the α7 helix; and Trp228 from the α8 helix form an extensive hydrophobic surface or “greasy palm” of the “cupped hand.” Lying loosely across this palm is the α6/α7 loop, which is itself very hydrophobic (Figure 3B). The loop is striking in that it lacks any clear secondary structure, yet it very neatly folds in on itself via van der Waal's interactions between hydrophobic residues. Residues Ile181, Trp183, Trp189, Pro193, Phe196, and Trp198 form a hydrophobic groove within which sits Leu207 and Leu209. The extensive hydrophobicity and intricate arrangement of residues suggest that this loop forms a stable structure that would resist unraveling.

In contrast to the intraloop packing, only two residues (Pro208 and Leu210) protrude from the α6/α7 loop into the “greasy palm” of the GUN4 core domain's “cupped hand.” Mutation of either position to alanine leads to the expression of misfolded protein as determined by inclusion body production in E. coli during attempted purification of recombinant samples (unpublished data). The necessity of both Pro208 and Leu210 in maintaining protein stability is not surprising, given the lack of interaction between the α6/α7 loop and the “cupped hand.” Analysis of this region of the structure reveals that the juncture of the α6/α7 loop with the “greasy palm” forms an extended hydrophobic surface that is shielded by the “thumb” (α2 helix) and the “middle finger” (α3 and α4 helices). In total, this structural design forms a cavity that is hydrophobic in nature with a volume of about 5,000 Å^3^. Additionally, analysis of 2Fo-Fc electron density maps contoured at 1σ reveals several well-ordered water molecules within the confines of the greasy palm, which, given the high degree of hydrophobicity of this space, are unusual.

### Mapping the Porphyrin Binding Site

In an effort to determine the binding site for porphyrin within SynGUN4, we used NMR to analyze the full length protein in the absence and presence of Deutero. Comparison of spectra obtained from ^1^H-^15^N transverse relaxation-optimized spectroscopy (TROSY) experiments of SynGUN4 in the absence and presence of 1–2 mM Deutero reveals several shifting peaks that were picked with the program CARA (Figure 4A) [28]. Chemical shifts were calculated for those peaks whose position changes in the presence of Deutero. Partial sequence-specific assignment for the backbone atoms of shifting peaks was obtained using TROSY-type triple resonance, and ^15^N-resolved [^1^H,^1^H] nuclear overhauser effect spectroscopy (NOESY) experiments with a ^2^H, ^13^C, ^15^N-labeled sample of SynGUN4 (1–233) (Figure 4B).

**Figure 4:**
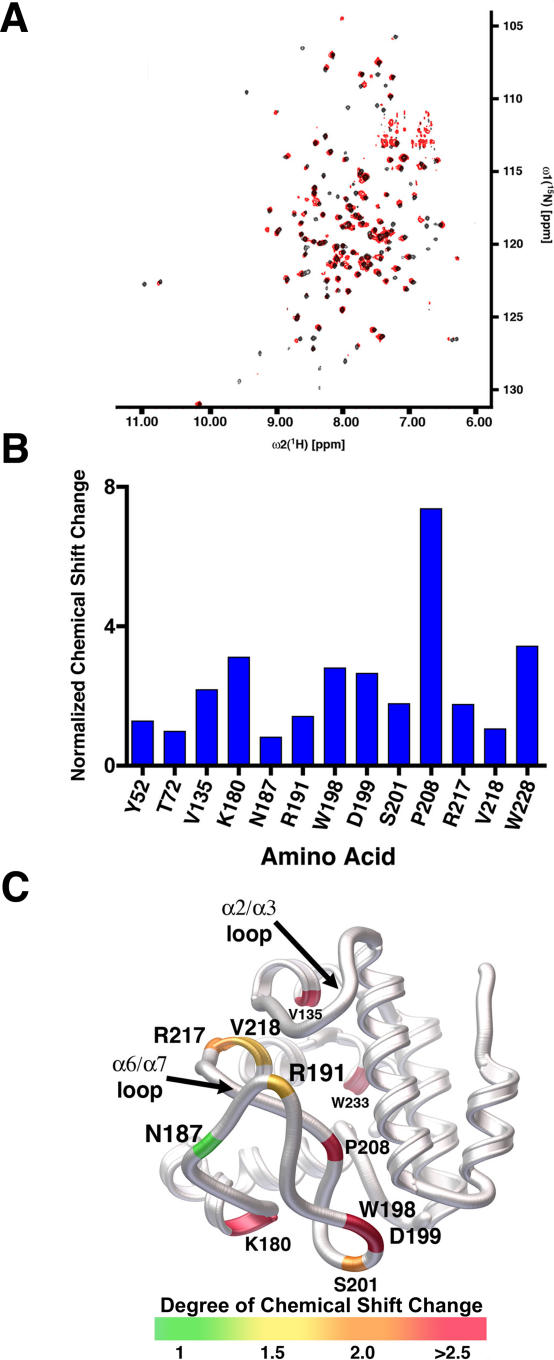
Analysis of SynGUN4 by NMR (A) Comparison of spectra obtained from ^1^H-^15^N TROSY experiments of SynGUN4 in the absence (black) and presence (red) of 2 mM deuteroporphyrin. (B) Normalized chemical shifts for those ^1^H-^15^N cross peaks whose positions change in the presence of 2 mM deuteroporphyrin. In general, the largest shifts cluster for residues on the α6/α7 loop. The remaining positions with significant chemical shifts reside on the “greasy palm” region of SynGUN4. (C) Rendered ribbon diagram of the Gun4 core domain with the position of the shifting ^1^H-^15^N cross peaks mapped onto the backbone structure of SynGUN4. The magnitude of the chemical shift changes shown corresponds to the color bar at the bottom. Briefly, shifts larger than 2.5 parts per million (ppm) are shown in red, shifts between 2 and 2.5 ppm are shown in orange, shifts between 1.5 and 2 ppm are shown in yellow, and shifts of 1.5 ppm and less are shown in green.

The vast majority of the residues whose environments drastically change are within the GUN4 core domain (Figure 4C). Of the 13 residues that were found to exhibit significant chemical shift perturbations in the presence of Deutero, only two (Tyr59 and Thr72) are located within the nonconserved N-terminal helical bundle domain. Of the remaining residues, three residues (Val135, Val218, and Trp228) form part of the “greasy palm,” six residues (Asn187, Arg191, Trp198, Asp199, Ser201, and Pro208) are localized within the α6/α7 loop, and the remaining two residues (Lys180 and Arg217) form the hinge regions at the ends of the α6/α7 loop. The chemical shifts offer a very precise measure of the chemical environment around the backbone nitrogen atoms at these positions. The observed chemical shift changes upon addition of Deutero suggest that the α6/α7 loop is undergoing a significant conformational change and/or that an electron-rich molecule, such as Deutero, lies within close proximity. As mentioned earlier, the interaction between this α6/α7 loop and the hydrophobic “greasy palm” of the GUN4 core domain is not extensive, and rearrangements of either or both necessary to accommodate the porphyrin molecule are feasible in both a dynamic and architectural sense. Taken together, the NMR data and the crystal structure strongly suggest that the porphyrin binding region lies within the pocket formed at the intersection of the α6/α7 loop and the “greasy palm” of the GUN4 core domain's “cupped hand” region.

### Deuteroporphyrin IX and Mg-Deuteroporphyrin IX Binding

First, the affinity of wild-type SynGUN4 for various porphyrins was investigated (Figure 5A). The dissociation constants (*K*_d_) for protoporphyrin analogs, Mg-Deutero (0.449 ± 0.045 μM) and Deutero (0.865 ± 0.146 μM) were measured. Additionally, SynGUN4's dissociation constants for deuteroporphyrin IX 2,4-(4,2) hydroxyethyl-vinyl-(deutero-divinyl) (3.94 ± 0.739 μM), hemin (4.73 ± 1.16 μM), N-methyl mesoporphyrin IX (NMMP) (11.0 ± 0.673 μM) and cobalt (III) protoporphyrin IX (Co-Proto) (2.67 ± 0.856 μM) were determined. SynGUN4 displayed the highest affinity for Mg-Deutero and Deutero with a preference for the metal-bound porphyrin. The reported dissociation constants for the *Synechocystis* Mg-chelatase enzyme, ChlH, are 1.22 ± 0.420 μM for Deutero and 2.43 ± 0.460 μM for Mg-Deutero [29].

**Figure 5:**
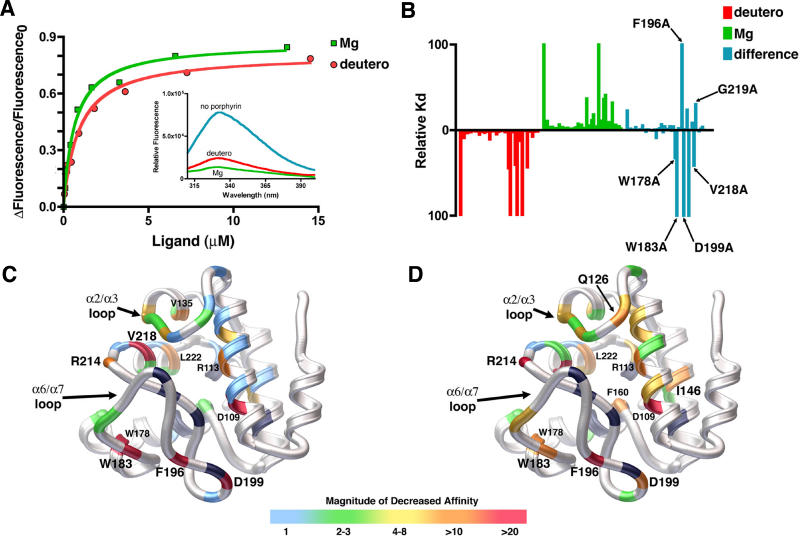
Quantitative Analysis of Porphyrin Binding by SynGUN4 (A) Comparison of the binding of SynGUN4 to analogs of both Proto and Mg-Proto. Both Mg-Deutero and Deutero quench endogenous tryptophan fluorescence upon binding (inset). A single binding site was assumed for the fitted line. (B) Relative dissociation constants were determined for each mutant and compared to the wild-type dissociation constant for both Deutero (red bars) and Mg-Deutero (green bars). The difference between these two sets of constants was calculated (blue bars). (C) Rendered ribbon diagram of the GUN4 core domain with the relative dissociation constants of each mutant for Deutero mapped onto the structure. While in some cases several different amino acid replacements were tested at particular positions, only the results obtained for the alanine mutations are mapped on the backbone structure shown. The x-fold change in the magnitude of the affinity of Deutero for each mutant is color-coded, as depicted by the scale shown at the bottom. Most amino acid changes did not alter binding affinity, as shown by the preponderance of light blue. Of the mutations that measurably alter binding affinity, the majority reside on the “greasy palm” of the GUN4 core domain. Several other energetic hotspots reside on the α2/α3 and α6/α7 loops. Positions of mutations that exhibit a greater than 10-fold decrease in affinity are labeled. Positions colored black failed to produce properly folded protein when mutated to alanine and expressed in E. coli. (D) Rendered ribbon diagram of the GUN4 core domain with the relative dissociation constants of each mutant for Mg-Deutero mapped onto the structure. Color coding is the same as for (C). In contrast to Deutero binding, many more mutants alter in vitro binding as shown by the lesser amount of light blue and the prominence of green and yellow color coding.

Analysis of SynGUN4's affinity for other porphyrins provides a larger context from which to deduce the determinants of binding specificity. Both deuteroporphyrins are smaller than the other porphyrins examined in that they lack two ethylene groups. Hemin and Co-Proto, which closely mimic the size of the naturally occurring substrate Mg-Proto, bind with slightly weaker affinities. This suggests that Mg-Proto and Proto may also bind with slightly weaker affinity then the their Deutero versions. Nonetheless, both hemin and Co-Proto bind as well as deutero-divinyl, which accurately mimics the size of the naturally occurring metal free Proto. This further supports that the less flexible metal bound porphyrins are indeed high affinity ligands for SynGUN4. Interestingly, NMMP, which mimics distorted porphyrins binds with very weak affinity. NMMP is a potent inhibitor of enzymes known to catalyze insertion of metals into porphyrins, including ferrochelatase [21,30]. SynGUN4, however, appears to favor the more planar metal-bound porphyrins.

To quantify the energetic contributions of specific residues in SynGUN4 involved in porphyrin binding, a series of putative porphyrin-binding site mutants were made and dissociation constants were measured. Most of the side chains were mutated to alanine; however, at some positions, other amino acids were investigated to explore the importance of electrostatics, side chain volume, or hydrophobicity in greater detail. Endogenous tryptophan fluorescence quenching of SynGUN4 by Deutero and Mg-Deutero was used to determine the *K*_d_ for all point mutants for which protein could be expressed and purified (Table 2). Relative values of all the dissociation constants for each mutant were determined by comparing the *K*_d_ of each mutant to that measured for wild-type SynGUN4 (Figure 5B).

**Table 2:**
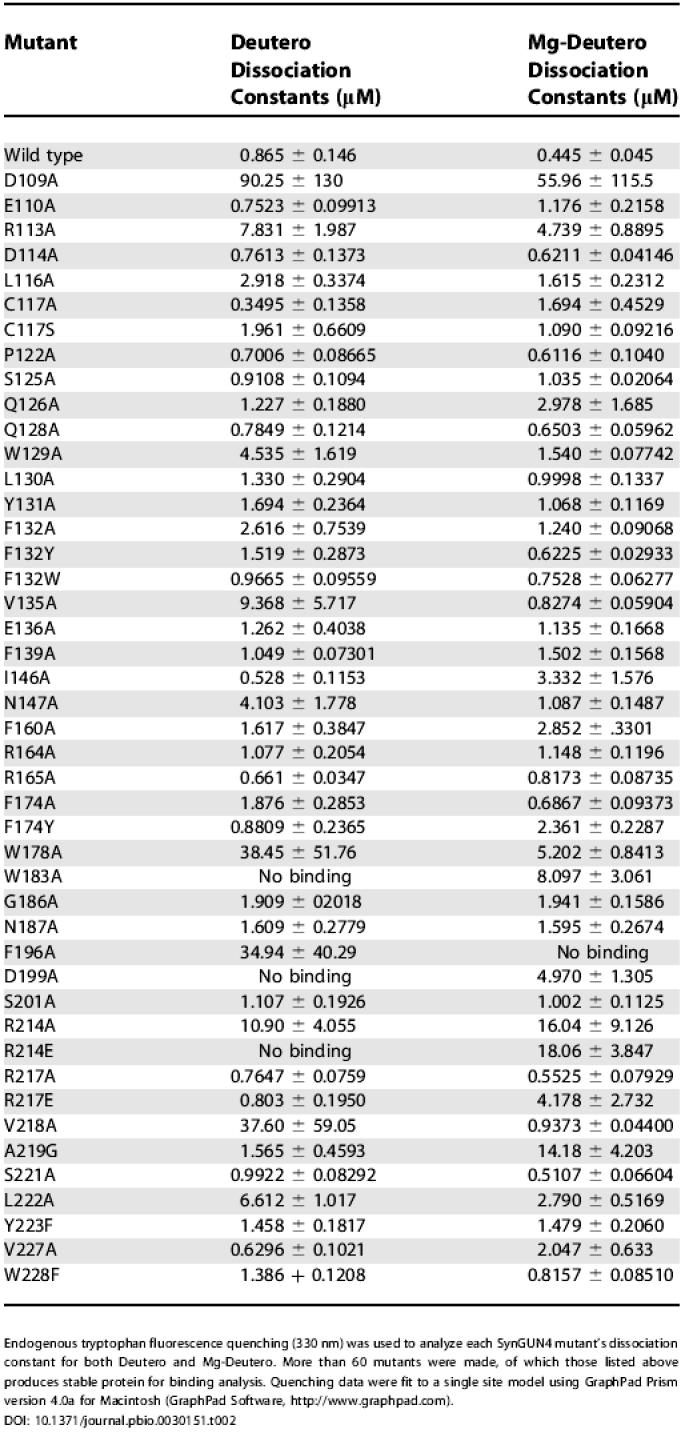
Dissociation Constants of SynGUN4 Proteins for Deutero and Mg-Deutero

Endogenous tryptophan fluorescence quenching (330 nm) was used to analyze each SynGUN4 mutant's dissociation constant for both Deutero and Mg-Deutero. More than 60 mutants were made, of which those listed above produces stable protein for binding analysis. Quenching data were fit to a single site model using GraphPad Prism version 4.0a for Macintosh (GraphPad Software, http://www.graphpad.com)

Comparison of the relative values for binding to both Deutero and Mg-Deutero shows that the majority of positions affecting binding cluster in neighboring regions of the GUN4 core domain. The positions that appear to be most critical for binding to both porphyrins reside in the α2/α3 and α6/α7 loops, as well as on the faces of the α2, α3, and α7 helices that form significant parts of the “greasy palm” within the “cupped hand” of SynGUN4. Calculation of the difference between the two sets of the relative *K*_d_ values shows that certain residues appear to be more important for binding Deutero instead of Mg-Deutero, and vice versa. For example, Val135 and Val218 appear to be essential for Deutero binding but not for Mg-Deutero binding. Alternatively, Ser125, Gln126, Ile146, Phe160, and A219 appear to be essential for specifically involved in binding Mg-Deutero.

Comparison of Mg-Deutero binding to Deutero binding shows that binding of the metal-bound porphyrin is much more sensitive to changes within the GUN4 core domain (Figure 5C and 5D). This difference most likely reflects the highly specialized architecture involved in binding the more rigid Mg-Deutero.

### Modeling of Porphyrin Binding

Armed with an energy map of relevant positions for porphyrin binding, we generated a model of SynGUN4 bound to Mg-Proto (Figure 6). In this model, the porphyrin molecule sits over Leu210 within the α6/α7 loop, deep in the “greasy palm” of SynGUN4. The solvent-exposed section of the α6/α7 and α2/α3 loops bracket the porphyrin, burying it deep within SynGUN4 core domain. Significantly, the carboxyl moieties of the porphyrin insert between Arg214 and Arg217, which would be complementary to the charge of the carboxylic acid groups extending from the porphyrin scaffold. Analysis of the Bacillus subtilis ferrochelatase structure bound to NMMP shows that this porphyrin-binding chelatase uses a pair of conserved arginines to bind the carboxyl moieties extending off the porphyrin scaffold (Figure 6A). The Arg214 and Arg217 positions within the α6/α7 loop of SynGUN4 closely resemble the arginine motif found on ferrochelatase, suggesting that this motif in SynGUN4 may function in an analogous fashion upon porphyrin binding (Figure 6A).

**Figure 6:**
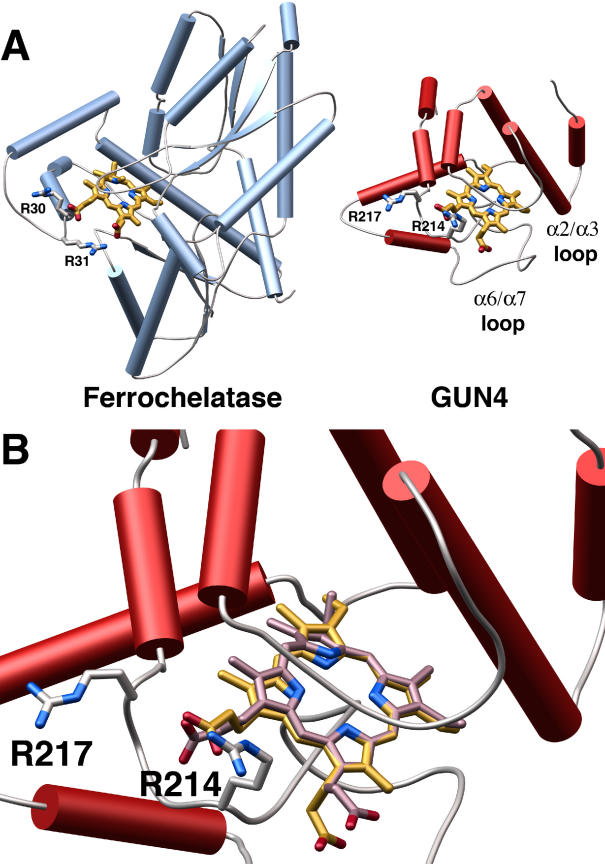
Model of a Putative SynGUN4 Porphyrin Complex Compared to an Experimentally Determined Structure for Ferrochelatase Bound to NMMP (A) Comparison of the crystal structure of the B. subtilis ferrochelatase bound to NMMP to the model of the SynGUN4 core domain bound to Mg-Proto. The SynGUN4 core domain • Mg-Proto model was generated by GOLD [54]. The carboxylic acid moieties of the porphyrin were staggered between the δ-guanido side chains of Arg214 and Arg217. The position of the arginine loop used to tether the carboxyl moieties of the porphyrin bound to ferrochelatase served as the fixed point for the structural alignment of SynGUN4 and ferrochelatase. (B) Close-up view of the structural alignment between Mg-Proto (gold) and NMMP (lavender). Attempts to strictly superimpose all of the atoms of the two porphyrins resulted in at least one corner of the porphyrin scaffold residing out of the plane defined by the flat Mg-Proto complex, because of the pucker of NMMP.

Arg214 is absolutely essential for binding to both porphyrins, with both alanine and glutamate substitutions completely abolishing binding (Table 2). Sequence comparisons of plant and bacterial GUN4s show that Arg214 is highly conserved. *Synechocystis* 6803 contains two genes that encode proteins related to *Arabidopsis* GUN4, previously named SynGUN4b and SynGUN4c, that are less similar to *Arabidopsis* GUN4 than is SynGUN4 [20]. The SynGUN4b sequence has an asparagine at the Arg214 position, which could functionally substitute for the arginine. The SynGUN4c sequence has a tyrosine at this position, however. This leads us to propose that SynGUN4c does not efficiently bind to Proto or Mg-Proto, but rather may interact with other cellular metabolites.

An interesting difference between Deutero and Mg-Deutero binding is seen at the Arg217 position. Alanine substitution at this position has no effect on either Deutero or Mg-Deutero binding. However, glutamate substitution at this position drastically reduces affinity for only Mg-Deutero by as much as 15-fold. The Arg217 is not as highly conserved, frequently being replaced by glutamine. The glutamine substitution, however, retains the overall aliphatic nature of the arginine side chain, as well as an amide available for interactions with the carboxyl moiety of Proto or Mg-Proto.

In an effort to address this difference, NMMP and Mg-Proto were structurally aligned in the putative SynGUN4 porphyrin binding pocket. Analysis of the NMMP molecule shows that one of its corners lies below the plane of the other three. An effort to model NMMP onto Mg-Proto always results in at least one of the four corners of the NMMP molecule lying outside the flattened plane of the porphyrin structural alignment. If the corners that are most buried within the protein are aligned and held fixed, this superpositioning produces a noticeable difference in the position of the carboxyl moieties between the metal-bound and metal-free porphyrin (Figure 6B). Specifically, the position of the carboxyl moiety closest to Arg217 is altered. We propose that the metal-free porphyrins most likely can avoid the electrostatic conflict produced by substituting Arg217 with glutamate through its ability to ruffle or pucker. The metal-bound porphyrin's enforced rigidity does not allow it to avoid electrostatic conflict with the glutamate, which results in a loss of binding affinity. These observations collectively suggest that, although Arg217 is not essential for binding to Mg-Proto, it nonetheless plays some role in selectivity of planar porphyrins.

### Enhancing Mg-Chelatase Activity

The effect of SynGUN4 on the activity of the Mg-chelatase was next investigated. Stoichiometric amounts of SynGUN4 dramatically increased the rate of Mg^2+^ incorporation into Deutero, as determined by an increase in fluorescence emission of Mg-Deutero (Figure 7A). The apparent Michaelis constant (*K*_m_) for Deutero was 7.46 ± 3.24 μM in a Mg-chelatase reaction containing 200 nM ChlH. The measured *K*_m_ for Deutero corresponded well with that previously reported for *Synechocystis* 6803 Mg-chelatase [29]. In Mg-chelatase reactions containing SynGUN4, the apparent *K*_m_ for Deutero was 1.48 ± 0.371 μM at 200 nM SynGUN4, and 1.65 ± 0.357 μM at 400 nM SynGUN4.

**Figure 7:**
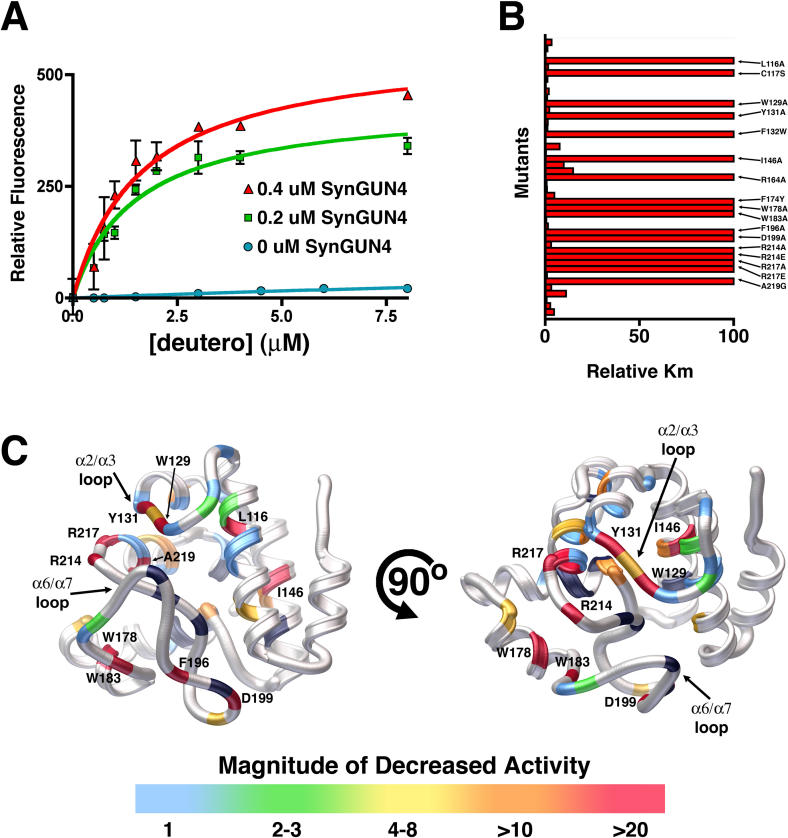
Quantitative Analysis of the GUN4 Stimulation of Mg^2+^ Incorporation into Deutero (A) The *K*_m_ values for GUN4 assisted Mg-Deutero biosynthesis were determined using both 0.2 μm (red) and 0.4 μM SynGUN4 (green). The resultant values contrast with those obtained for Mg^2+^ incorporation by the Mg-chelatase complex in the absence of SynGUN4 (blue). (B) Relative *K*_m_ values (compared to wild-type SynGUN4) were determined for each SynGun4 mutant previously examined for Deutero binding. (C) Rendered ribbon diagrams of orthogonal views of the SynGUN4 core domain with the relative *K*_m_ values of each SynGUN4 mutant mapped onto the structure. The color-coded scale for each mutation's effect on Mg^2+^ incorporation is shown at the bottom. Several mutants that altered Mg^2+^ incorporation activity but previously did not affect binding to deuteroporphyrin IX were uncovered including a R217A mutation and the Trp129 and Tyr131 residing on the α2/α3 loop. Mutants of Val218, the later of which is critical for binding Deutero but not for binding Mg-Deutero showed no effect on chelatase activity while mutants of Ala219, the later of which is essential for binding to Mg-Deutero, completely failed to stimulate Mg-chelatase activity. Only those mutant SynGUN4s exhibiting a greater than 10-fold change in *K*_m_ are labeled. Shown in black are residues that, when mutated to alanine, failed to produce properly folded protein upon expression in E. coli.

Interestingly, the Mg-chelatase complex from *Rhodobacter sphaeroides,* which carries out anoxygenic photosynthesis and lacks a GUN4 homolog, has a much lower *K*_m_ than that measured for *Synechocystis* Mg-chelatase. This seems to suggest that the development of GUN4 activity coincided with a decrease in the basal catalytic efficiency of the Mg-chelatase complex in the absence of GUN4. Oxygenic phototrophs may require a porphyrin-binding protein such as GUN4 to minimize oxidative stress induced by free Proto and Mg-Proto in the presence of O_2_ and bright light. Mg-Proto shielding may be a critical event in the evolution of photosynthesis in oxygen-rich environments.

Next, each point mutant previously tested for Deutero and Mg-Deutero binding was assayed for its ability to enhance Mg^2+^ incorporation carried out by the Mg-chelatase complex (Figure 7B). Measurements at several time points were analyzed, and the resulting values were compared to the *K*_m_ determined for SynGUN4 enhancement of Mg^2+^ incorporation. Many of the mutants that had no energetic effect on porphyrin binding also did not dramatically affect SynGUN4's stimulatory activity on the holo Mg-chelatase. Several mutants did demonstrate significant effects on enzymatic activity, however. Many of the mutants, not surprisingly, are clustered in the α6/α7 loop, including residues that are essential for binding porphyrin (Figure 7C). Specifically, a region of the α2/α3 loop (Trp129, Leu130, and Tyr131) appears to be essential for Mg^2+^ incorporation. This loop region is moderately important for porphyrin binding as well.

No mutants deficient in Mg-Proto binding enhanced Mg-chelatase complex activity. These data support a mechanism in which porphyrin binding by SynGUN4 underlies its stimulatory effect on the Mg-chelatase complex. A mechanism in which SynGUN4 stimulates the Mg-chelatase complex by assisting with product release, as opposed to localizing substrates, is further supported by the fact that the Val218 position, which is essential for binding to Deutero but not for Mg-Deutero, had no effect on Mg^2+^ incorporation. The Ala219 position, which is essential for Mg-Deutero binding, did have a significant effect on stimulation of Mg-chelatase activity. Conversely, the Ala219 position is not essential for binding to the metal-free porphyrin.

Nonetheless, some mutants that had only moderate to no effect on binding appear to dramatically alter enzymatic activity. Among these are two residues (Cys117 and Ile146) within the putative porphyrin binding pocket; one residue (Phe132) on the α3 helix; and Arg217. Serine substitution at the Cys117 position only moderately affects porphyrin binding while completely abolishing Mg-chelatase activity. This cysteine lies directly behind the α2/α3 loop, which has been shown here to be critical for GUN4-assisted Mg^2+^ incorporation activity. The role of the sulfhydryl group in stabilizing loop rearrangements is not fully understood, but clearly, substitution of a hydroxyl group at this position does not suffice to replace wild-type activity.

Interestingly, mutation of Phe132 to alanine moderately affects binding to both porphyrins. Mutation to tyrosine and tryptophan restores normal binding activity. Nonetheless, the tryptophan mutation at this position has severely compromised enzymatic activity. This suggests that this section of SynGUN4 may modulate interactions with the Mg-chelatase complex. The more bulky hydrophobic groups may help to shield the Arg217-porphyrin interaction from the solvent. However, a too-large side chain at this position, such as a tryptophan, may prevent the GUN4 • Mg-chelatase interactions necessary to assist in product release from the chelatase enzyme. Finally, the Arg217 substitution to alanine abolishes GUN4's stimulatory effect on the Mg-chelatase complex while having no apparent effect on binding of either Deutero or Mg-Deutero to GUN4. This suggests that Arg217 plays a role in the selection of chelatase products that is dependent on the presence of the chelatase enzymes.

## Discussion

Mg-Proto accumulation is a plastid signal that regulates the expression of a large number of nuclear genes encoding proteins localized to the chloroplast [6]. GUN4 is a recently discovered Mg-Proto and Mg-chelatase subunit binding protein that appears to participate in plastid-to-nucleus signaling and chlorophyll synthesis in *Arabidopsis* [20]. Here, we describe the novel three-dimensional structure of SynGUN4, an *Arabidopsis* GUN4 homolog, as well as the porphyrin binding properties of GUN4. Three *GUN4* relatives in *Synechocystis* 6803*,* previously named *SynGUN4a* (referred to as *SynGUN4* in this paper), *SynGUN4b,* and *SynGUN4c* [20], correspond to loci *sll0558, sll1380,* and *slr1958,* respectively [31]. In contrast, *GUN4* is a single-copy gene in plant genomes [20]. Because the amino acid sequence derived from *SynGUN4/sll0558* is more similar to *Arabidopsis* GUN4 than are those derived from the other two *Synechocystis* 6803 *GUN4* relatives, it was concluded that *SynGUN4/sll0558* was most likely the ortholog of *Arabidopsis GUN4* [20]. Recently, a null allele of *SynGUN4/sll0558* was reported to cause a severe chlorophyll-deficient phenotype, while a null allele of *SynGUN4b/sll1380* was reported to have no effect on chlorophyll levels [23]. An *Arabidopsis* null mutant, *gun4–2,* does not accumulate chlorophyll under normal growth conditions [20], which suggests that *SynGUN4/sll0558* is the true ortholog of *Arabidopsis GUN4.* Therefore, SynGUN4 residues that are important for porphyrin binding and Mg-chelatase stimulation are likely to be important in plant GUN4 proteins as well.

GUN4 appears to play a role in photoprotection, since *Arabidopsis* plants containing the null allele, *gun4–2,* accumulate chlorophyll only in very dim light conditions [20]. Our results are consistent with GUN4 having a photoprotective role within the chloroplast. Biophysical and biochemical data support a model wherein the hydrophobic “greasy palm” of the GUN4 core domain, formed by helices that comprise the fingers of the “cupped hand,” wrap around the porphyrin molecule, potentially shielding it from the bulk solvent of the plant or bacterial cell. Our model suggests that porphyrins are buried deep within the GUN4 core domain, where they would be protected from collisions with molecular oxygen. The unique “cupped hand” fold of the GUN4 core domain could serve as a novel vehicle for shuttling porphyrin within the chloroplast while inhibiting cellular damage caused by porphyrin-mediated generation of reactive oxygen species formed in the presence of bright light and oxygen.

In addition to shielding porphyrin from the oxygen-rich environment of the cell, GUN4 also appears to be a necessary component of the Mg-chelatase in oxygenic photosynthetic organisms. For example, in *Rhodobacter capsulatus,* which carries out anoxygenic photosynthesis, bacteriochlorophyll synthesis is rapidly shut down in the presence of molecular oxygen and bright light by a mechanism that involves both gene repression and Mg-chelatase inhibition [21]. Under these conditions, the largest subunit of R. capsulatus Mg-chelatase, BchH, forms covalent adducts with bound Proto, which inactivates the enzyme [32]. Species that perform anoxygenic photosynthesis do not appear to contain *GUN4*-like genes [20]. The largest subunit of the Mg-chelatase complex from both *Rhodobacter* (BchH) and *Synechocystis* (ChlH) bind to both Mg-Proto and Proto, as well as to their analogs, Mg-Deutero and Deutero. However, in contrast to ClhH, BchH binds to the metal-free porphyrin (Deutero) with higher affinity than to Mg-Deutero. It is possible that GUN4 enables ChlH to bind Mg-Proto tightly, like BchH, while simultaneously providing photoprotective functions required for species that carry out oxygenic photosynthesis.

Distortion of metal-bound porphyrin molecules requires more energy (65–130 kJ/mole) than is needed to distort metal-free porphyrins (48 kJ/mole) [33]. In ferrochelatase, once the metal ion is inserted, the porphyrin becomes much more difficult to bend, leading to a decrease in ferrochelatase affinity and ultimately leading to metallized porphyrin release. Conversely, GUN5 maintains some affinity for its metal-containing porphyrin products, creating a potentially deleterious situation in which inhibitory adducts form under conditions of oxygenic photosynthesis. This energetic observation suggests a mechanism in which GUN4 assists in product release from the Mg-chelatase enzyme through its preference for the more planar and rigid Mg-Proto species. In turn, GUN4 may prevent the formation of covalent adducts between the largest subunit of the Mg-chelatase and bound porphyrins in oxygenic photosynthetic organisms by stimulating product release while additionally protecting the Mg-chelatase enzyme active site from molecular oxygen and/or bright light. The mechanism in which SynGUN4 stimulates the Mg-chelatase complex by assisting with product release is supported by the fact that the Val218 position, which is essential for binding to Deutero but not Mg-Deutero had no effect on Mg^2+^ incorporation, whereas the Ala219 position, which is essential for Mg-Deutero binding, did have a significant effect on stimulation of Mg-chelatase activity.

The question arises as to how GUN4 discriminates between different metal-bound porphyrins. Quantum mechanical calculations predict that the energy required to distort porphyrin bound to Fe^2+^ and Mg^2+^ are nearly equivalent [33]. The implication of this calculation is that both metals distort the porphyrin scaffold equally and confer comparable rigidity upon the tetrapyrrole once either metal cation is inserted. The biophysical models, and the observation that GUN4 binds porphyrins containing different metal ions with different affinities, intimate the existence of a mechanism mediating preferential selection of specific metal-bound porphyrins based on the bound cation rather than on any structural differences exerted by the metal onto the porphyrin scaffold. Although we were unable to test Mg-Proto directly because of its decreased solubility (unpublished data), we were able to determine that Co-Proto binds with higher affinity than hemin. This in vitro experiment further suggests that, indeed, there may be some intrinsic difference between metal-bound porphyrins that SynGUN4 is able to distinguish. Copurification with GUN5 and the lower *K*_d_ for Mg-Deutero suggest that GUN4 functions specifically in Mg-porphyrin biosynthesis and trafficking, but currently we cannot rule out a role for GUN4 in the biosynthesis and trafficking of porphyrins that contain other metals (e.g., heme).

Other porphyrin-binding proteins have been described, among them the mammalian mitochondrial (peripheral-type) benzodiazepine receptor (PBR) and the tryptophan-rich sensory protein (TspO) from R. sphaeroides [34,35,36,37]. PBR is found in most tissues but is expressed at high levels in steroid-synthesizing tissues such as the liver, where it is thought to act as a cholesterol transporter in steroidogenesis [38,39,40]. Interestingly, Proto has been shown to inhibit binding of the high affinity PBR agonist 1-(2-chlorophenyl)-N-methyl-(1-methylpropyl)-3-isoquinoline carboxamide (PK 11195) [36]. Additionally, E. coli cells expressing PBR uptake Proto, and this activity can be inhibited by cholesterol [41]. Another Proto-binding protein, TspO, has been demonstrated to negatively regulate the transcription of specific photosynthetic genes in R. sphaeroides 2.4.1 [37,42]. The physiological significance of Proto binding to these proteins is not fully understood, although both proteins are believed to be involved in tetrapyrrole homeostasis.

TspO and PBR are highly related to one another [43]. Interestingly, sequence alignment of these proteins with GUN4 reveals 40% similarity to TspO and 44% similarity to PBR (see Figure 2B). What is notable from the sequence alignment is that the homology is clustered in the helical regions. More specifically, many of the residues that constitute the “greasy palm” of the cupped hand of the GUN4 core domains are conserved. Additionally, the Arg214 position is conserved, which is consistent with the purported ability of PBR and TspO to bind to Proto. Both of these proteins are thought to exist as integral membrane transport proteins consisting of five helices. Indeed, only four of the five predicted helical regions show homology to those of GUN4. Notably, the homologous regions are critically involved in porphyrin binding in GUN4. Additionally, PBR and TspO lack the α7 and α8 helices seen in SynGUN4, which, if absent, would create a significant pore through the GUN4 architecture. This pore might represent an essential feature of the transport functions of PBR and/or TspO.

The GUN4 core domain fold presented here describes the structure of a dedicated noncatalytic porphyrin-binding protein. Moreover, the combined use of NMR and x-ray crystallography viewed in the context of subsequent quantitative analysis of porphyrin binding allowed us to propose a mechanism for porphyrin sequestration by GUN4 and GUN4-like proteins. Together, these structural and energetic analyses form a solid foundation for understanding GUN4 activity, and they add to our understanding of the role of GUN4 in the stimulation of Mg-chelatase activity, as well as in Mg-Proto retrograde signaling.

## Materials and Methods

### 

#### Cloning, mutagenesis, and purification

The SynGUN4 used in these studies was isolated from *Synechocystis* genomic DNA [20] and was subcloned into the NcoI and BamHI restriction sites in the pHIS8–3 vector [44]. E. coli strain BL21 (DE3) was transformed (Stratagene, La Jolla, California, United States) and cells were grown at 37 °C to an OD_600_ of 0.8. Expression was induced by adding IPTG (Boehringer Mannheim, Amsterdam, Netherlands) to 0.1 mM, and cells were allowed to grow for an additional 6 h at 20 °C. Bacteria were harvested by centrifugation (8,000 × *g*) at 5 °C; cell pellets were stored at −70 °C. Cell pellets were thawed and resuspended in 50 mM Tris-Cl (pH 8.0), 500 mM NaCl, 10 mM imidazole (pH 8.0), 10% (v/v) glycerol, 1% (v/v) Tween-20, and 10 mM β-mercaptoethanol (β-ME) (Sigma, St. Louis, Missouri, United States) at 4 °C. Resuspended cells were sonicated, and lysates were cleared by centrifugation (100,000 × *g*) at 4 °C. His-tagged SynGUN4 was purified from clarified supernatants by Ni^2+^-chelation chromatography (Qiagen, Valencia, California, United States). Bound protein was eluted in 50 mM Tris-Cl (pH 8.0), 500 mM NaCl, 250 mM imidazole (pH 8.0), 10% (v/v) glycerol, and 10 mM β-ME. The N-terminal octa-histidine tag was removed by thrombin (Sigma) digestion during dialysis against 50 mM Tris-Cl (pH 8.0), 500 mM NaCl, and 20 mM β-ME at 4 °C for 24 h. The sample was further purified over a Superdex 200 26/60 gel filtration column (Amersham Biosciences, Little Chalfont, United Kingdom) equilibrated in dialysis/thrombin cleavage buffer. Peak fractions were collected and dialyzed against 5 mM Tris-Cl (pH 8.0) containing 10 mM β-ME, concentrated to 30 mg/ml using an Amicon Centricon 10 column (Millipore, Billerica, Massachusetts, United States), and stored at −70 °C.

All site-directed mutants of SynGUN4 used for activity assays were constructed using the QuikChange (Stratagene) protocol. Wild type and mutants for binding and activity assays were expressed using pHIS8–3 and purified as described above, except the thrombin cleavage step was eliminated. NMR samples were prepared with aforementioned expression procedures in minimal media containing 4 g of L^−1^ ^13^C-D-glucose and 1 g of L^−1^ ^15^N-ammonium sulfate (Cambridge Isotopes Laboratories, Andover, Massachusetts, United States). Protein purification was carried out as described [45]. A final yield of ^13^C, ^15^N-labeled *Synechocystis* GUN4 at a final concentration of 0.6 mM was obtained from 2 l of expression media.

#### Crystallization and structure determination

Crystals of SynGUN4 were grown by the hanging drop vapor diffusion method at 4 °C by mixing 1.0 μl of SynGUN4 (30 mg/ml) with 1.0 μl of a reservoir solution containing 7% PEG 8,000 (Sigma), 200 mM NaBr, 50 mM HEPES-Na^+^ (pH 7.5), and 2 mM DTT. Crystals were stabilized in 15% (v/v) ethylene glycol, 5% (v/v) PEG 8,000, 200 mM NaBr, 50 mM HEPES-Na^+^ (pH 7.5), and 2 mM DTT, and rapidly frozen in a 100-K stream of nitrogen gas. Native (2.2 Å) and derivative data were collected on a MacScience Imaging plate detector, DIP 2020k (MacScience Corporation, Yokohama, Japan), using double focusing Pt/Ni coated mirrors and CuKα X-rays from a MacScience M18XHF generator operating at 4.5 kW (50 kV and 90 mA). Data were processed with DENZO and SCALEPACK [46]. The crystals contain one molecule per asymmetric unit (52.9% solvent) and belong to the space group P2_1_2_1_2_1_ (a = 64.21 Å, b = 71.19 Å, c = 72.89 Å; α = β = γ = 90.0°). Hg and Pt sites from crystals soaked in 1 mM methyl mercury (II) chloride (Hg) and 1 mM potassium tetrachloroplatinate (II) (Pt), respectively, were located and used for phasing with SOLVE/RESOLVE [47]. The figure of merit (FOM) was 0.3 after MIRAS phasing and 0.60–0.67 after density modification [48]. Automated model building was carried out in SOLVE/RESOLVE [49]. The SOLVE/RESOLVE-generated model was manually improved using the experimental electron density maps displayed in O [50]. The resulting model was positionally refined against all of the Hg data, using the default bulk solvent model in CNS with maximum likelihood targets [51]. The model consists of residues 1–233 of SynGUN4 and 249 water molecules. PROCHECK [52] revealed a total of 91% of the residues in the most favored region of the Ramachandran plot and 9% in the additionally allowed region. Main chain and side chain structural parameters were consistently better than average (overall *G* value of 0.46).

#### NMR experiments

All of the NMR spectra were recorded at 22 °C on a Bruker 700-MHz spectrometer equipped with four radiofrequency channels and a triple-resonance cryoprobe with shielded *z*-gradient coil. The NMR samples contained 0.6 mM ^13^C, ^15^N-labeled SynGUN4 in 5 mM Tris-Cl (pH 8.0), 300 mM NaCl, 1 mM DTT/95% H_2_O/5% D_2_O. Partial sequence assignment and structure determination were performed with the standard protocol for ^13^C, ^15^N-labeled samples [53]. ^1^H, ^13^C, and ^15^N backbone resonances were assigned using the triple-resonance experiments HNCA and CBCA(CO)NH and three-dimensional ^15^N-resolved [^1^H, ^1^H]-NOESY experiments. Chemical-shift perturbation experiments were carried out with NMR samples in the absence and presence of 2 mM deuteroporphyrin dissolved in 5 mM Tris-Cl (pH 8.0).

#### Binding analysis

Endogenous tryptophan quenching of SynGUN4 was measured using fluorescence spectroscopy as described previously [29]. Briefly, serial dilutions of both Deutero and Mg-Deutero were made in DMSO. SynGUN4 was diluted into 50 mM MOPS-KOH (pH 7.7), 150 mM NaCl, and 1 mM DTT. Fluorescence quenching data were collected on a PTI Alphascan spectrofluorimeter (Photon Technology Instruments, Santa Clara, California) by monitoring 200 nM GUN4 tryptophan fluorescence at 332 nm. Dissociation equilibrium constants for GUN4-porphyrin interactions were determined by fitting titration data to a one-site binding model.

#### Modeling

The program GOLD was used to dock the Mg-Proto in the GUN4 core domain of SynGUN4 [54]. A 16-Å cavity was defined around the carbon atom of the terminal methyl group of residue Leu210 in the crystal structure of SynGUN4. The majority of outputs from GOLD were similar to each other in the position of features such as the porphyrin carboxyl moieties. Minor manual adjustments of the GOLD solution were made using the programs Chimera [55] and O [51].

#### Mg-chelatase activity

Mg-chelatase subunits were expressed and purified as previously described [20,56]. Reactions contained indicated concentrations of SynGUN4. Concentrations of the Mg-chelatase components used were 0.1 μM ChlH, 0.1 μM ChlD and 0.2 μM ChlI, which were similar to those used previously [56]. Reactions were carried out using Deutero, as previously described, in 50 mM MOPS-KOH (pH 7.7), 20 mM MgCl_2_, 1 mM DTT, and 5 mM ATP [20]. Each 100-μl reaction mixture was incubated at 34 °C and stopped by addition of 900 μl of acetone/water/32% (v/v) ammonia (80:20:1, by volume) [56]. Reactions were then centrifuged at 15,000 × *g* for 5 min at room temperature. The Mg-Deutero concentration was determined by fluorescence spectroscopy. The *K*_m_ was determined by analysis of the rise in the slope of Mg-Deutero synthesis at several different starting substrate concentrations using the program PRISM 4.0 (GraphPad Software, San Diego, California, United States).

## Supporting Information

### Accession Numbers

Coordinates for *Synechocystis* GUN4 (accession number 1Y6I) have been deposited in the Protein Data Bank (http://www.rcsb.org).

## Abbreviations

NMMP: N-methyl mesoporphyrin
NMR: nuclear magnetic resonance
PBR: peripheral-type benzodiazepine receptor
TROSY: transverse relaxation-optimized spectroscopy
TspO: tryptophan-rich sensory protein
ppm: parts per million

## References

[pbio-0030151-b01] Butow RA, Avadhani NG (2004). Mitochondrial signaling: The retrograde response. Mol Cell.

[pbio-0030151-b02] Zhang K, Kaufman RJ (2004). Signaling the unfolded protein response from the endoplasmic reticulum. J Biol Chem.

[pbio-0030151-b03] Rodermel S (2001). Pathways of plastid-to-nucleus signaling. Trends Plant Sci.

[pbio-0030151-b04] Richly E, Dietzmann A, Biehl A, Kurth J, Laloi C (2003). Covariations in the nuclear chloroplast transcriptome reveal a regulatory master-switch. EMBO Rep.

[pbio-0030151-b05] Rodermel S, Park S (2003). Pathways of intracellular communication: Tetrapyrroles and plastid-to-nucleus signaling. Bioessays.

[pbio-0030151-b06] Strand Å, Asami T, Alonso J, Ecker JR, Chory J (2003). Chloroplast to nucleus communication triggered by accumulation of Mg-protoporphyrin IX. Nature.

[pbio-0030151-b07] Johanningmeier U, Howell SH (1984). Regulation of light-harvesting chlorophyll-binding protein mRNA accumulation in Chlamydomonas reinhardtii. Possible involvement of chlorophyll synthesis precursors. J Biol Chem.

[pbio-0030151-b08] Oster U, Brunner H, Rüdiger W (1996). The greening process in cress seedlings. V. Possible interference of chlorophyll precursors, accumulated after thujaplicin treatment, with light-regulated expression of Lhc genes. Photochem Photobiol.

[pbio-0030151-b09] Kropat J, Oster U, Rüdiger W, Beck CF (1997). Chlorophyll precursors are signals of chloroplast origin involved in light induction of nuclear heat-shock genes. Proc Natl Acad Sci U S A.

[pbio-0030151-b10] Kropat J, Oster U, Rüdiger W, Beck CF (2000). Chloroplast signalling in the light induction of nuclear HSP70 genes requires the accumulation of chlorophyll precursors and their accessibility to cytoplasm/nucleus. Plant J.

[pbio-0030151-b11] La Rocca N, Rascio N, Oster U, Rüdiger W (2001). Amitrole treatment of etiolated barley seedlings leads to deregulation of tetrapyrrole synthesis and to reduced expression of Lhc and RbcS genes. Planta.

[pbio-0030151-b12] Chen JJ, London IM (1995). Regulation of protein synthesis by heme regulated eIF-2 alpha kinase. Trends Biochem Sci.

[pbio-0030151-b13] Zhang L, Hach A (1999). Molecular mechanism of heme signaling in yeast: The transcriptional activator Hap1 serves as the key mediator. Cell Mol Life Sci.

[pbio-0030151-b14] Ogawa K, Sun J, Taketani S, Nakajima O, Nishitani C (2001). Heme mediates derepression of Maf recognition element through direct binding to transcription repressor Bach1. EMBO J.

[pbio-0030151-b15] Qi Z, O'Brian M (2002). Interaction between the bacterial iron response regulator and ferrochelatase mediates genetic control of heme biosynthesis. Mol Cell.

[pbio-0030151-b16] Cornah JE, Roper JM, Pal Singh D, Smith AG (2002). Measurement of ferrochelatase activity using a novel assay suggests that plastids are the major site of haem biosynthesis in both photosynthetic and non-photosynthetic cells of pea *(Pisum sativum L*.*)*. Biochem J.

[pbio-0030151-b17] Thomas J, Weinstein JD (1990). Measurement of heme efflux and heme content in isolated developing cotyledons. Plant Physiol.

[pbio-0030151-b18] Mochizuki N, Brusslan JA, Larkin R, Nagatani A, Chory J (2001). *Arabidopsis* genomes uncoupled 5 (GUN5) mutant reveals the involvement of Mg-chelatase H subunit in plastid-to-nucleus signal transduction. Proc Natl Acad Sci U S A.

[pbio-0030151-b19] Susek RE, Ausubel FM, Chory J (1993). Signal transduction mutants of *Arabidopsis* uncouple nuclear *CAB* and *RBCS* gene expression from chloroplast development. Cell.

[pbio-0030151-b20] Larkin RM, Alonso JM, Ecker JR, Chory J (2003). GUN4, a regulator of chlorophyll synthesis and intracellular signaling. Science.

[pbio-0030151-b21] Willows RD (2003). Biosynthesis of chlorophylls from protoporphyrin IX. Nat Prod Rep.

[pbio-0030151-b22] Whyte BJ, Castelfranco PA (1993). Breakdown of thylakoid pigments by soluble proteins of developing chloroplasts. Biochem J.

[pbio-0030151-b23] Wilde A, Mikolajczyk S, Alawady A, Lokstein H, Grimm B (2004). The GUN4 gene is essential for cyanobacterial porphyrin metabolism. FEBS Lett.

[pbio-0030151-b24] D'Andrea LD, Regan L (2003). TPR proteins: The versatile helix. Trends Biochem Sci.

[pbio-0030151-b25] Yaffe MB, Elia AE (2001). Phosphoserine/threonine-binding domains. Curr Opin Cell Biol.

[pbio-0030151-b26] Schleiff E, Soll J (2000). Travelling of proteins through membranes: Translocation into chloroplasts. Planta.

[pbio-0030151-b27] Holm L, Sander C (1998). Touring protein fold space with Dali/FSSP. Nucl Acids Res.

[pbio-0030151-b28] Keller R, Groudinsky O, Wuthrich K (1976). Contact-shifter resonances in the 1H NMR spectra of cytochrome b5. Resonance identification and spin density distribution in the heme group. Biochim Biophy Acta.

[pbio-0030151-b29] Karger GA, Reid JD, Hunter CN (2001). Characterization of the binding deuteroporphyrin IX to the magnesium chelatase H subunit and spectroscopic properties of the complex. Biochemistry.

[pbio-0030151-b30] Lecerof D, Fodje M, Hansson A, Hansson M, Al-Karadaghi S (2000). Structural and mechanistic basis of porphyrin metallation by ferrochelatase. J Mol Biol.

[pbio-0030151-b31] Kaneko T, Sato S, Kotani H, Tanaka A, Asamizu E (1996). Sequence analysis of the genome of the unicellular cyanobacterium *Synechocystis* sp. strain PCC6803. II. Sequence determination of the entire genome and assignment of potential protein-coding regions. DNA Res.

[pbio-0030151-b32] Willows RD, Lake V, Roberts TH, Beale SI (2003). Inactivation of Mg chelatase during transition from anaerobic to aerobic growth in Rhodobacter capsulatus. J Bacteriol.

[pbio-0030151-b33] Sigfridsson J, Ryde U (2003). The importance of porphyrin distortions for the ferrochelatase reaction. Biol Inorg Chem.

[pbio-0030151-b34] Hirsch JD, Beyer CF, Malkowitz L, Loullis CC, Blume AJ (1988). Characterization of ligand binding to mitochondrial benzodiazepine receptors. Mol Pharmacol.

[pbio-0030151-b35] Kessel D (1988). Interactions between porphyrins and mitochondrial benzodiazepine receptors. Cancer Lett.

[pbio-0030151-b36] Verma A, Snyder S (1988). Characterization of porphyrin interactions with peripheral type benzodiazepine receptors. Mol Pharmacol.

[pbio-0030151-b37] Yeliseev AA, Kaplan S (1999). A novel mechanism for the regulation of photosynthesis gene expression by the TspO outer membrane protein Rhodobacter sphaeroides 2.4.1. J. Biol Chem.

[pbio-0030151-b38] Papadopoulos V, Mukhin AG, Costa E, Krueger KE (1990). The peripheral-type benzodiazepine receptor is functionally linked to Leydig cell steroidogenesis. J Biol Chem.

[pbio-0030151-b39] Anholt RR, De Souza EB, Oster-Granite ML, Snyder SH (1985). Peripheral-type benzodiazepine receptors: Autoradiographic localization in whole-body sections of neonatal rats. J Pharmacol Exp Ther.

[pbio-0030151-b40] Anholt RR, Aebi U, Pedersen PL, Snyder SH (1986). Solubilization and reassembly of the mitochondrial benzodiazepine receptor. Biochemistry.

[pbio-0030151-b41] Wendler G, Lindemann P, Lacapere JJ, Papadopoulos V (2003). Protoporphyrin IX binding and transport by recombinant mouse PBR. Biochem Biophys Res Commun.

[pbio-0030151-b42] Zeng X, Kaplan S (2001). TspO as a modulator of the repressor/antirepressor (PpsR/AppA) regulatory system in Rhodobacter sphaeroides 2.4.1. J Bacteriol.

[pbio-0030151-b43] Yeliseev AA, Kaplan S (2000). TspO of rhodobacter sphaeroides. A structural and functional model for the mammalian peripheral benzodiazepine receptor. J Biol Chem.

[pbio-0030151-b44] Jez JM, Ferrer JL, Bowman ME, Dixon RA, Noel JP (2000). Dissection of malonyl-coenzyme A decarboxylation from polyketide formation in the reaction mechanism of a plant polyketide synthase. Biochemistry.

[pbio-0030151-b45] Perrin MH, DiGuccio MR, Koerber SC, Rivier JE, Kunitake KS (2003). A soluble form of the first extracellular domain of mouse type 2beta corticotropin-releasing factor receptor reveals differential ligand specificity. J Biol Chem.

[pbio-0030151-b46] Otwinowski Z, Minor W (1997). Processing of X-ray diffraction data collected in oscillation mode. Macromol Crystallogr, Pt A.

[pbio-0030151-b47] Terwilliger TC, Berendzen J (1999). Automated MAD and MIR structure solution. Acta Crystallogr D Biol Crystallogr.

[pbio-0030151-b48] Terwilliger TC (2000). Maximum likelihood density modification. Acta Crystallogr D Biol Crystallogr.

[pbio-0030151-b49] Terwilliger TC (2002). Automated main-chain model-building by template-matching and iterative fragment extension. Acta Crystallogr D Biol Crystallogr.

[pbio-0030151-b50] Jones AT, Zou J-Y, Cowan S, Kjeldgaard M (1991). Improved methods for building protein models in electron density maps and the location of errors in these models. Acta Crystallogr A.

[pbio-0030151-b51] Brunger AT, Adams PD, Clore GM, DeLano WL, Gros P (1998). Crystallography and NMR system: A new software suite for macromolecular structure determination. Acta Crystallogr D Biol Crystallogr.

[pbio-0030151-b52] Laskowski RA, MacArthur MW, Moss DS, Thornton JM (1993). PROCHECK: A program to check the stereochemical quality of protein structures. J Appl Crystallogr.

[pbio-0030151-b53] Cavanagh J, Fairbrother WJ, Palme AG, Skelton NJ (1996). Protein NMR spectroscopy, principles and practice.

[pbio-0030151-b54] Jones G, Willet P, Glen RC, Leach AR, Taylor R (1997). Development and validation of a genetic algorithm for flexible docking. J Mol Biol.

[pbio-0030151-b55] Pettersen EF, Goddard TD, Huang CC, Couch GS, Greenblatt DM (2004). UCSF chimera—A visualization system for exploratory research and analysis. J Comput Chem.

[pbio-0030151-b56] Jensen PE, Gibson LCD, Hunter CN (1998). Determinants of catalytic activity with the use of purified I, D and H subunits of the magnesium protoporphyrin IX chelatase from *Synechocystis* PCC6803. Biochem J.

[pbio-0030151-b57] Kraulis PJ (1991). MOLSCRIPT. A program to produce both detailed and schematic plots of protein structures. J Appl Crystallogr.

[pbio-0030151-b58] Nicholls A, Sharp KA, Honig B (1991). Protein folding and association: Insights from the interfacial and thermodynamic properties of hydrocarbons. Proteins.

